# Supplementary material for: Correlation of clinical decision-making with probability of disease: A web-based study among general practitioners
Source: PLoS One. 2020 Oct 29;15(10):e0241210. doi: 10.1371/journal.pone.0241210 (PMC7595298; doi:10.1371/journal.pone.0241210)
Supplement: S1 Text — (DOCX) [file pone.0241210.s001.docx]

**S1 Text. Example of a pharyngitis vignette (corresponding to a McIsaac score of 4).**

A 32 year-old woman presents with a 3-day history of sore throat. She has no cough, dyspnea, ear pain or runny nose.

On physical examination, temperature is 38.2 °C (without antipyretics), blood pressure is 118/70 mmHg, pulse rate is 86/min and respiration rate is 18/min. The pharynx is erythematous with tonsillar enlargement and exudates. The neck shows several sensitive bilateral lymphadenopathies. The chest is clear to auscultation.
