# Supplementary material for: Correlation of clinical decision-making with probability of disease: A web-based study among general practitioners
Source: PLoS One. 2020 Oct 29;15(10):e0241210. doi: 10.1371/journal.pone.0241210 (PMC7595298; doi:10.1371/journal.pone.0241210)
Supplement: S1 Table — (PDF) [file pone.0241210.s002.pdf]

Total Alvarado Score	Real probability of having appendicitis [20, 21]
1	<39%
2	42 - 53%
3	44 - 55%
4	47 - 58%
5	50 - 64%
6	57 - 71%
7	61 - 79%
8	62 - 86%
9	66 - 99%
**Total McIsaac Score**	**Real probability of having pharyngitis [22]**
0	1 - 2,5%
1	5 - 10%
2	11 - 17%
3	28 - 35%
4	51 - 53%
5	>53%
