# Supplementary material for: Correlation of clinical decision-making with probability of disease: A web-based study among general practitioners
Source: PLoS One. 2020 Oct 29;15(10):e0241210. doi: 10.1371/journal.pone.0241210 (PMC7595298; doi:10.1371/journal.pone.0241210)
Supplement: S2 Table — (PDF) [file pone.0241210.s003.pdf]

| Management options proposed for pharyngitis | Corresponding categories<br><br>***A) No investigations, no treatment → disease ruled out***<br>***B) Obtain more information by ordering a test → test required (= more investigation)***<br>***C) Treat empirically with no further diagnostic testing → disease ruled in*** |
|---|---|
| Symptomatic treatment, no further investigation | A |
| Obtain a rapid streptococcal antigen test and treat only patients who test positive | B |
| Obtain a throat culture and treat only patients with positive culture | B |
| Treat empirically but obtain a throat culture and stop the treatment if the culture is negative | C |
| Treat the disease empirically with antibiotics | C |
| Other | A, B or C according to the free answers |

| Management options proposed for Appendicitis | Corresponding categories<br><br>***A) No investigations, no treatment → disease ruled out***<br>***B) Obtain more information by ordering a test → test required (= more investigation)***<br>***C) Treat empirically with no further diagnostic testing → disease ruled in*** |
|---|---|
| Reassurance and discharge | A |
| Reassessment in 24-48h (watchful waiting and reassessment) | A |
| Obtain an US | B |
| Obtain a CT | B |
| Admit to hospital | C |
| Give antibiotics immediately (to treat the beginning of an appendicitis) and reassessment in 24-48h | C |
| Refer to surgeon | C |
| Other | A, B or C according to the free answers |
