# Supplementary material for: Correlation of clinical decision-making with probability of disease: A web-based study among general practitioners
Source: PLoS One. 2020 Oct 29;15(10):e0241210. doi: 10.1371/journal.pone.0241210 (PMC7595298; doi:10.1371/journal.pone.0241210)
Supplement: S3 Table — (PDF) [file pone.0241210.s004.pdf]

	Appendicitis (n = 574)			Pharyngitis (n = 574)		
	Weighted kappa (95%CI)			Weighted kappa (95%CI)		
	0.74 (0.70 – 0.78)			0.66 (0.62 – 0.71)		
Sex of physician	Male	Female	p of the difference	Male	Female	p of the difference
	0.77	0.70	0.091	0.66	0.69	0.438
Country	Swiss	USA		Swiss	USA	
	0.75	0.73	0.755	0.61	0.76	<0.001
Age of physician	<50	>50		<50	>50	
	0.75	0.76	0.695	0.76	0.57	<0.001
Sex of patient	Male	Female		Male	Female	
	0.76	0.72	0.367	0.66	0.66	0.970
