# Supplementary material for: Correlation of clinical decision-making with probability of disease: A web-based study among general practitioners
Source: PLoS One. 2020 Oct 29;15(10):e0241210. doi: 10.1371/journal.pone.0241210 (PMC7595298; doi:10.1371/journal.pone.0241210)
Supplement: S4 Table — (PDF) [file pone.0241210.s005.pdf]

	Appendicitis (n = 574)			Pharyngitis (n = 574)		
	Weighted kappa (95%CI)			Weighted kappa (95%CI)		
	0.61 (0.56 – 0.66)			0.70 (0.66 – 0.73)		
Sex of physician	Male	Female	p of the difference	Male	Female	p of the difference
	0.60	0.65	0.563	0.71	0.68	0.412
Country	Swiss	USA		Swiss	USA	
	0.63	0.56	0.217	0.64	0.81	<0.001
Age of physician	<50	>50		<50	>50	
	0.65	0.57	0.152	0.75	0.64	0.005
Sex of patient	Male	Female		Male	Female	
	0.59	0.63	0.407	0.74	0.65	0.019
