# Supplementary material for: Correlation of clinical decision-making with probability of disease: A web-based study among general practitioners
Source: PLoS One. 2020 Oct 29;15(10):e0241210. doi: 10.1371/journal.pone.0241210 (PMC7595298; doi:10.1371/journal.pone.0241210)
Supplement: S5 Table — aUnderestimation, bCorrect estimation, cOverestimation. (PDF) [file pone.0241210.s006.pdf]

		Appendicitis (n = 559)				Pharyngitis (n = 559)			
		Under[a]	Correct[b]	Over[c]	p-value	Under	Correct	Over	p-value
		**61%**	**29%**	**10%**		**22%**	**39%**	**39%**	
**Sex of physician**	**Male**	62%	27%	11%	0.258	25%	38%	37%	0.042
	**Female**	58%	33%	9%		16%	42%	42%	
**Country**	**Swiss**	60%	29%	12%	0.337	22%	38%	40%	0.500
	**USA**	63%	29%	8%		21%	43%	36%	
**Age of physician**	**<50**	63%	28%	9%	0.420	20%	41%	40%	0.459
	**>50**	58%	30%	12%		24%	38%	38%	
**Sex of patient**	**Male**	61%	28%	11%	0.843	24%	38%	38%	0.396
	**Female**	61%	29%	10%		20%	41%	40%	
